# Spoligotyping of Clinical Isolates of *Mycobacterium tuberculosis* Complex Species in the Oromia Region of Ethiopia

**DOI:** 10.3389/fpubh.2022.808626

**Published:** 2022-03-17

**Authors:** Bedru Hussien, Aboma Zewude, Biniam Wondale, Awraris Hailu, Gobena Ameni

**Affiliations:** ^1^Department of Public Health, Goba Referral Hospital, Madda Walabu University, Goba, Ethiopia; ^2^Malaria and Neglected Tropical Diseases Research Team, Ethiopian Public Health Institute, Ministry of Health, Addis Ababa, Ethiopia; ^3^Department of Veterinary Medicine, College of Agriculture and Veterinary Medicine, United Arab Emirates University, Al Ain, United Arab Emirates; ^4^Department of Biology, Arba Minch University, Arba Minch, Ethiopia; ^5^Department of Public Health, College of Health Sciences, Debre Birhan University, Debre Birhan, Ethiopia; ^6^Aklilu Lemma Institute of Pathobiology, Addis Ababa University, Addis Ababa, Ethiopia

**Keywords:** *Mycobacterium tuberculosis*, spoligotyping, clustering, orphan, singleton, Ethiopia, shared type

## Abstract

**Background:**

Tuberculosis (TB) is a leading cause of morbidity and mortality in Ethiopia. Investigation of the *Mycobacterium tuberculosis* complex (MTBC) species circulating in the Ethiopian population would contribute to the efforts made to control TB in the country. Therefore, this study was conducted to investigate the MTBC species and spoligo patterns in the Oromia region (central) of Ethiopia.

**Methods:**

A cross-sectional study design was used to recruit 450 smear positive pulmonary TB (PTB) cases from the Oromia region between September 2017 and August 2018. Mycobacteria were isolated from sputum samples on the Lowenstein Jensen (LJ) medium. Molecular identification of the isolates was performed by spoligotyping. The results of spoligotyping were transferred into a query box in the SITVIT2 database and Run TB-Lineage in the TB Insight website for the identification of spoligo international type (SIT) number and linages of the isolates, respectively. Statistical Product and Service Solutions (SPSS) 20 was applied for statistical analysis.

**Results:**

Three hundred and fifteen isolates were grouped under 181 different spoligotype patterns. The most dominantly isolated spoligotype pattern was SIT149 and it consisted of 23 isolates. The majority of the isolates were grouped under Euro-American (EA), East-African-Indian (EAI), and Indo-Oceanic (IO) lineages. These lineages consisted of 79.4, 9.8, and 9.8% of the isolates, respectively. One hundred and sixty-five of the isolates were classified under 31 clustered spoligotypes whereas the remaining 150 were singleton types. Furthermore, 91.1% of the total isolates were classified as orphan types. Clustering of spoligotypes was associated (*p* < 0.001) with EAI lineage.

**Conclusion:**

SIT149 and EA lineage were predominantly isolated from the Oromia region substantiating the findings of the similar studies conducted in other regions of Ethiopia. The observation of significant number of singleton and orphan spoligotypes warrants for additional genetic typing of the isolates using method(s) with a better discriminatory power than spoligotyping.

## Introduction

Tuberculosis (TB) remained to be a serious public health problem worldwide because of its high incidence rate, coinfection with human immunodeficiency virus (HIV), and multidrug resistance (1–6). Globally, 10.0 million individuals developed TB disease in 2019; and 1.2 million HIV-negative individuals died due to TB, whereas 2,08,000 deaths occurred in people living with HIV (5). The global reduction rate in the incidence of TB was not as fast as 4–5% every year as required to achieve the initial stage of the end TB strategy by 2020 (6). Besides, the high prevalence of active TB, the incidence of latent TB is high, which exacerbates the overall burden of TB since latent progresses to active TB disease within a lifetime of the individuals. It is estimated that 1.7 billion (about 23%) of the world's population is infected with latent TB (3). One of the factors leading to the progression of latent TB to active TB disease is infection with HIV. For example, according to the 2020 WHO global TB report, the risk of developing TB in people living with HIV was about 18 times higher than in the rest of the global population (5). In this regard, the 2020 WHO global TB report indicated that 8.2% of the incident TB cases in 2019 occurred in individuals living with HIV (5). The proportion of TB cases coinfected with HIV was highest in countries of the WHO African Region, and it was more than 50% in some parts of southern Africa (5).

The rising number of resistant strains of *M. tuberculosis* worldwide imposes a serious challenge to the control of TB programs of nations. In 2020 alone, 71% of the 2.1 to 3.0 million bacteriologically confirmed pulmonary TB (PTB) cases recorded worldwide were rifampicin-resistant (6). Moreover, 1,57,903 of these cases were multidrug-resistant (MDR)–rifampicin-resistant (RR) whereas 25,681 of them were preextensive drug-resistant (XDR) or XDR (6), which underlines the threatening situation of drug resistance in TB. Pre-XDR TB cases are cases who infected with the MDR-TB strains that are resistant to either fluoroquinolones (FQs) or second-line injectable drug, but not both. On the other hand, XDR-TB cases are cases who infected with MDR-TB strains that are resistant to any FQs and one of the second-line injectable drugs (capreomycin, kanamycin, or amikacin) (6).

Ethiopia is located in the Horn of Africa with an estimated population of about 112 million living in a low socioeconomic status (7). The country is among the 30 high TB burden countries with the estimated incidence of 140 cases per 100,000 population in 2019, and 6.5% of these cases were coinfected with HIV (5). Furthermore, Ethiopia was one of the 30 high MDR/RR TB burden countries until 2020 (4, 6, 8). Fortunately, country has transitioned out of the list of 30 high MDR/RR-TB burden countries in 2021 (6). The estimated TB mortality rate in HIV-negative cases in 2019 in Ethiopia was estimated to be 19 per 100,000 population (5). The socioeconomic problems, including chronic malnutrition, overcrowding, and high prevalence of HIV infection, fueled the transmission of TB in the country (9). The transmission of *M. tuberculosis* from a TB patient to a contact person depends on exposure duration, intensity of exposure, cough, and sputum-related host factors and the virulence of *M. tuberculosis* strain (10).

Molecular typing of MTBC isolates has improved information on the epidemiology of TB and has assisted to advance TB control by providing information on transmission dynamics, external reinfection, investigating epidemics, and identifying the clonal spread of successful clones, including MDR ones (11, 12). The knowledge about the population of MTBC strains in a certain area is essential to understand the relationship between genotype and phenotype of MTBC strains that can help the TB control program. Spoligotyping is one of the molecular biological methods that is used to describe the genetic variety of MTBC and thus plays a great role in identifying the spoligotype patterns, the isolates of MTBC species in humans and animals (13–16).

Previous studies about molecular typing in different sites of Ethiopia have revealed the circulation of major lineages and clades of MTBC species, including Indo-Oceanic, East Asian/Beijing, East African-Indian, Euro-American, and lineage 7 (Ethiopian) (17, 18). Euro-American was the most frequently isolated lineage in the country whereas East Asian was the least frequently isolated lineage in the country (17–22). The Ethiopian lineage also called lineage 7 seemed to be common to northeastern Ethiopia (18). The most common clades identified in the country were T, CAS, H, Manu, and Ethiopian whereas the predominant SIT numbers were SIT149, SIT53, SIT25, SIT37, and SIT21 (23).

Differences in genetic makeup of the MTBC strains render them a variety of biological and epidemiological phenotypes (10). These phenotypes are illustrated by characteristics, such as transmission potential, disease severity, and progression rate from infection to disease (11–13). For example, strains of lineages 2 and 4 are widely distributed (Europe, America, Africa, and East Asia); this suggests that the strains of these lineages could be more virulent than those limited to specific geographic regions, such as lineages 5 and 6 (west Africa) (10, 14, 15). Therefore, the identification of the spoligotypes and the lineages of MTBC species in the certain geographic region could inform the control program indirectly on the phenotypic characteristics of the isolate, so that the programs can consider the necessary actions. This study was conducted to investigate the spoligotypes of MTBC isolates circulating in the Oromia region of Ethiopia.

## Materials and Methods

### Study Area and Settings

**Figure 1:**
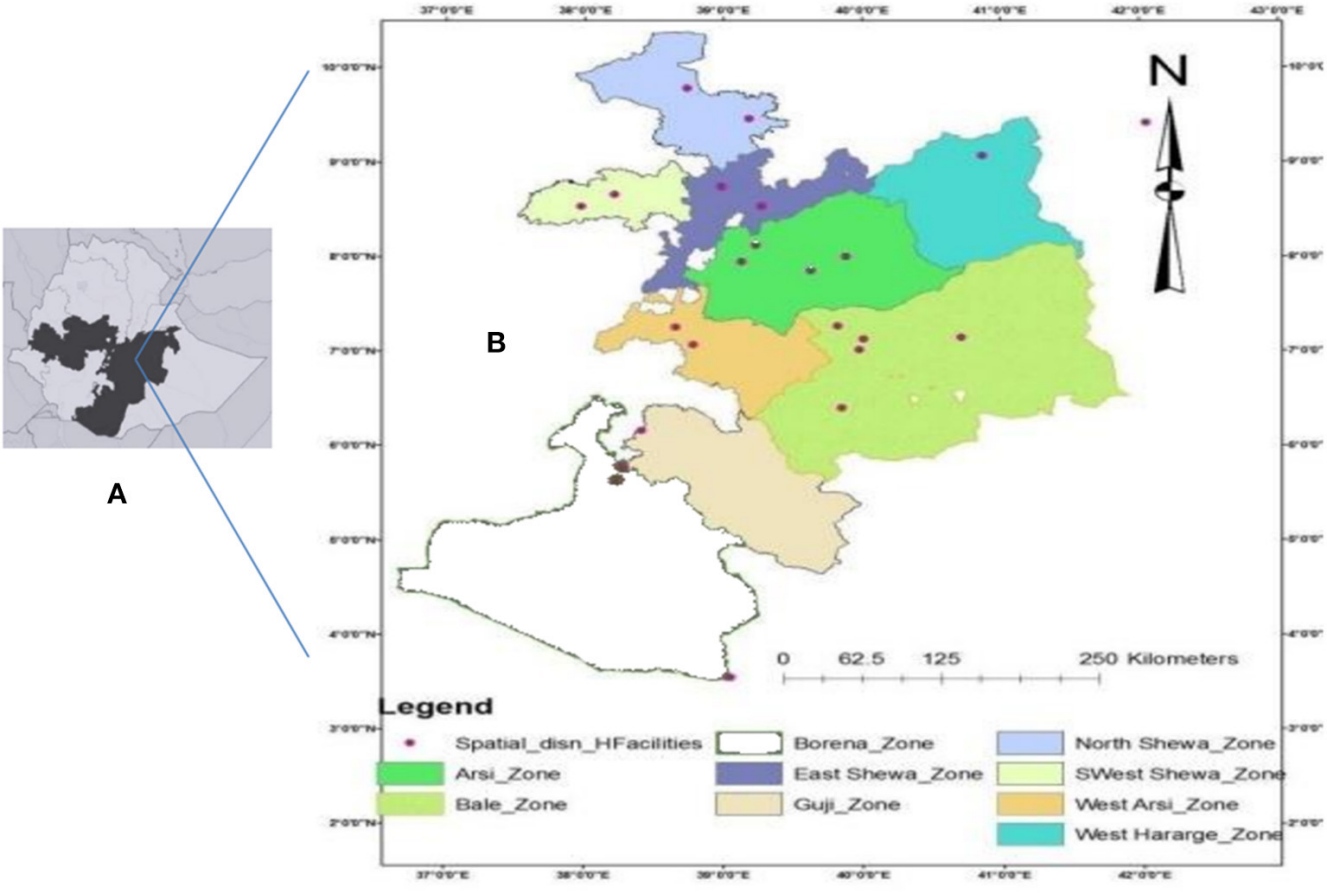
Map of Ethiopia showing Oromia Region, shaded **(A)** and Oromia Region showing Zones included in the study and spatial distribution of the health care facilities from where participants were sampled **(B)**.

The Oromia region is the largest regional state of Ethiopia accounting for about 45% of the total population of the country (Figure 1) and it owns 108 hospitals, 1,405 health centers, and 7,090 health posts that provide health service to its population (24). The Oromia region is known for its highest incidence and prevalence of TB as compared to the other regional states of the country. In this study, the patients were recruited from nine administrative zones of the 17 zones of the Oromia region. Sputum collection was conducted at the TB clinics of the health facilities located in the study zones. Mycobacterial isolation and genetic typing were performed at the Aklilu Lemma Institute of Pathobiology (ALIPB), the institute that undertakes considerable research in tropical and infectious diseases.

### Study Design and Patients

Health institution-based cross-sectional design was used for this study. Sputum samples were collected from 450 patients with TB during their visits to the health facilities for medical treatment. The samples were collected from individuals who were suspicious for PTB and referred to the TB clinics of the health centers and hospitals. These individuals were first examined by physicians on the outpatient department and then referred to TB clinics for submission of sputa samples. The sputum sample of each patient was shared between the research team and the health service providers.

### Sample Size and Sampling Procedure

The sample size was estimated to be 450 by considering the objective and the sample size of several local studies. Multistage sampling was used. That is, the zones of the region and Woredas (subzones) were selected based on their accessibility; health facilities were selected according to their accessibility, logistics, and previous history of TB patient flow. Thirty-two health facilities in nine zones were selected (Figure 1). For the study participants, a convenience sampling method was used whereby samples were taken from clients who came to the respective health facilities for medical consultation and were sequentially enrolled as they were diagnosed with PTB until the proportional estimated sample size was obtained at each health facility.

### Data Collection Instrument

Structured questionnaire was prepared, pre-tested and then used for data collection. In addition, clinical case forms were used for the capturing of clinical data of the patients.

### Sputum Collection and Sample Transportation

About 3 to 5 ml morning sputum samples were collected in a sterile plastic tube by the laboratory technicians for diagnosis as a part of the health service. Part of the sputum sample was spared for the research team (for this study). The sputum samples for this research purpose were stored in health facility laboratories for a maximum of 2 days in a temperature range of 2°C−8°C until being transported to the TB Laboratory of the ALIPB, Addis Ababa University. The transportation of the samples was made on ice pack carriers maintaining the cold chain. The preparation and packing of the specimen were according to the national standard operating procedures for biological sample transportation (25).

### Acid-Fast Staining and Culturing of Samples

The sputum samples that were processed for culturing were positive for acid-fast bacilli with Ziehl–Neelsen (ZN) staining procedure. The samples were cultured on (LJ) media according to the WHO guideline (26, 27). Briefly, the sputum sample was mixed with an equal volume of 4% NaOH and then centrifuged at speed of 3,000 g for 15 min for decontamination purposes. Neutralization of the sample was done by adding 10% HCl to the sediment of the sample and monitoring for the neutralization by dropping phenol red to the sediment. Neutralization was confirmed using a pH indicator. The neutralized sediment was inoculated onto two LJ media; one supplemented with glycerol and the other with pyruvate. Inoculated media were incubated at 37°C for up to 8 weeks. Mycobacterial growth was monitored every week. Culture was considered negative after 8 weeks if no growth was observed. Positive colonies were further confirmed by ZN staining. Heat treatment of mycobacterial isolates in dH_2_O at 80°C for 50 min was used for genomic DNA extraction without extensive DNA purification. Extracts were stored at −20°C until they were used for molecular characterization.

### Identification of *Mycobacterium tuberculosis* Complex

An immune-chromatographic assay (CapilaTM TB-Neo version 6.0. Tauns Laboratories, Inc. Japan) (28) was used to differentiate MTBC from other non-TB mycobacteria (NTM). The assay detects *M. tuberculosis* rapidly (in less than an hour) and accurately in positive cultures. Positivity to *M. tuberculosis* was indicated by the presence of red-purple color bands on both test (T) and control (C) areas of the test plate. Otherwise, it was considered as either negative (the presence of a band at only C) or invalid (the presence of a band at T but not at C). H37Rv (ATCC27294) was used as positive control for each test.

### Spoligotyping

Spoligotyping was performed as described by Kamerbeek et al. (29) and as per the spoligotype kit supplier's instructions (Ocimum Biosolutions, Ijsselstein, The Netherlands). DNA from known strains of *M. bovis* SB1176 and *M. tuberculosis* H37Rv was used as positive controls, whereas water (Qiagen company, Germany) was used as a negative control. Briefly, the direct repeat (DR) region was amplified using oligonucleotide primers (DRa: GGTTTTGGGTCTGACGAC) and DRb: CCGAGAGGGGACGGAAAC) derived from the DR sequence. The PCR product was denatured using a thermo-cycler at 96°C for 10 min. Then, the denatured product was hybridized by incubating for 60 minutes at 60°C to a set of 43 immobilized oligonucleotides, each corresponding to one of the unique spacer DNA sequences within the DR locus. After hybridization, the membrane was washed and then incubated in diluted streptavidin–peroxidase (HotStar, Crawley, UK) for 45–60 min at 42°C and then washed again. Then, DNA was detected by the enhanced chemiluminescence (ECL) method (Amersham, Biosciences, Amersham, UK) and by exposure to X-ray film (Hyperfilm ECL, Amersham) as specified by the manufacturer. The film was inserted into a film developer solution in a dark room after which it is moved to the fixer solution. Thereafter, the film was dried and ready for the interpretation of the result. The black squares were converted to 1, whereas the white squares were converted to 0 and then transferred to the SITVIT2 database query box for retrieving the SIT number. Isolates for which SIT could not be found in the SITVIT2 database were considered as orphans (30). Furthermore, the binary format of each isolate was converted to the octal forms in the SITVIT2 database, and then, the octal format was transferred to the Run TB-Lineage query box in the TB Insight website for identification of the lineages of the isolates (31, 32).

### Statistical Analysis

Descriptive statistics, including frequency of socio-demographic characteristics and clinical history, and frequency and distribution of major lineages or sub-lineages, was computed using SPSS 20, and outputs were presented using tables and figures. Binary logistic regression and multiple regression models were used to assess the presence of an association between major lineages or sublineages and selected sociodemographic characteristics (age and zone) plus clinical history (treatment history, status of BCG vaccination, and body mass index). Pearson's chi-square test was also used to assess the relationship between clustering status and zones, major lineages, and dominant strains. In spoligotyping, when a unique spoligotype pattern was exhibited by a single isolate, it was considered as a singleton whereas a spoligotype pattern that was exhibited by more than one isolate was considered as clustered type. Recent transmission index (RTI) was calculated using the formula RTI = (T(c) - N(c)) / T(p); where T(c) is the total number of clustered patients, N(c) is the total number of clusters, and T(p) is the total number of isolates (33). A spoligotype with a unique pattern that has not been found in the SITVIT2 database was defined as an orphan. On the other hand, a shared spoligotype was defined as a spoligotype pattern that had been registered in the SITVIT2 database.

### Ethics Approval and Consent to Participate

Ethical clearance was obtained from the Health Studies Higher Degree Committee of the University of South Africa (ref no: REC-012714-039, HSHDC/454/2015). Oromia Health Bureau, Ethiopia, permitted the fieldwork in the study area (ref no: BEFO/AHBTM/1-8/2308). The purpose of the study was explained to the study participants, and written consent or assent were obtained from each study participant.

## Results

### Sociodemographic Characteristics and Clinical History of the Study Participants

A total of 450 patients were included in the study, and the sociodemographic data of the study patients are presented in Table 1. More than two-third (68.7%) of the study patients were within the age group of 18–39 years with a fairly similar number of both sexes with a median age of 26 years. About 40% of the study participants were originated from the northern parts of the region and were daily laborers, jobless, or housewives. Additionally, more than half of them were from rural residents whereas one-fifth of them were previously treated as patients with PTB for at least 4 weeks. Moreover, a quarter of the patients had a history of Bacille Calmette–Guerin (BCG) vaccination although immunization of children with BCG is recommended by the Ethiopian Ministry of Health as a TB control strategy. TB-HIV coinfection was recorded in <10% of the patients (Table 1).

**Table 1:** Sociodemographic characteristics and clinical history of the study participants, *n* = 450, Oromia, Ethiopia, 2020.

**Characteristics**		**Number (%) of patients**	**95% Confidence interval (%)**
Age (years)	<18	52 (11.6)	8.4, 14.4
	18–28	203 (45.1)	40.7, 49.8
	29–39	106 (23.6)	19.6, 27.3
	40–45	55 (12.2)	9.3, 15.5
	>50	34 (7.6)	5.1, 10.2
Sex	Male	240 (53.3)	48.7, 58.2
	Female	210 (46.7)	41.8, 51.3
Address (Zones)	North Shewa	37 (8.2)	5.6, 10.9
	South West Shewa	28 (6.2)	4.0, 8.4
	East Shewa	92 (20.4)	16.9, 24.4
	Arsi	88 (19.6)	16.0, 23.1
	West Arsi	24 (5.3)	3.3, 7.6
	Bale	64 (14.2)	11.1, 17.3
	Guji	60 (13.3)	10.2, 16.4
	Borena	25 (5.6)	3.6, 7.6
	West Harerge	32 (7.1)	4.9, 9.6
Occupation	Farmer	160 (35.6)	30.9, 40.2
	Merchant	17 (3.8)	2.2, 5.8
	Government employee	10 (2.2)	1.1, 3.8
	Student	84 (18.2)	15.1, 22.2
	Others^*^	179 (39.8)	35.1, 44.2
Residence	Urban	174 (38.7)	34.2, 42.9
	Semi-urban	21 (4.7)	2.9, 6.7
	Rural	255 (56.7)	52.5, 60.9
Category of TB treatment	New	343 (76.2)	72.0, 80.0
	Previously treated	107 (23.6)	20.0, 28.0
HIV serum status	Negative	404 (89.8)	86.9, 92.7
	Positive	34 (7.6)	5.1, 10.0
	Unknown	12 (2.7)	1.3, 4.2
BCG vaccination status	Not vaccinated	322 (71.6)	66.9, 76.0
	Vaccinated	112 (24.9)	20.9, 29.1
	Do not remember	16 (3.6)	2.0, 5.6
Nutritional status	Severe under nutrition	40 (8.9)	6.7, 11.3
	Moderate under nutrition	52 (11.6)	8.7, 14.7
	Mild under nutrition	140 (31.1)	26.9, 35.1
	Normal	213 (47.3)	42.9, 52.0
	Overweight	5 (1.1)	0.2, 2.2

(*) *includes housewives, daily laborers, jobless, and underage; HIV, human immunodeficiency virus; BCG, Bacille Calmette–Guerin*.

### Molecular Typing Results

Culture positivity was 89.3% (402/450). Totally, six of the isolates were found to be NTM on identification whereas the spoligotype patterns for 81 isolates were poor and hence could not be interpreted. As a result, the spoligotype data of these 81 isolates were excluded from the analysis. A total of 181 spoligotype patterns were identified within 315 isolates. A total of 31 clustered spoligotype patterns were identified. These 31 clustered spoligotypes consisted of 165 isolates sharing 52.4% of the total isolates. The size of each cluster ranges from 2 to 23 isolates; SIT149 being the most dominant clustered spoligotype with 23 isolates (Table 2). In this study, the RTI was calculated to be 0.42.

**Table 2:** The spoligotype patterns of clustered spoligotypes or strains (*n* = 31) and their corresponding lineages or sublineages, Oromia, Ethiopia, 2020.

**SIT**	**Isolates with similar pattern**	**CBN lineage**	**SITVIT2 lineage/ sublineages**	**Octal number**	**Binary format**
149	23	EA	T3-ETH	777000377740661	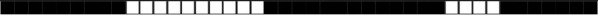
Orphan	18	EA	T3-ETH	557000077740261	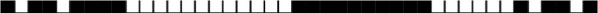
Orphan	12	IO	MANU2	557347577743661	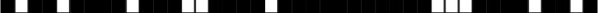
Orphan	9	IO	MANU3	777767777770771	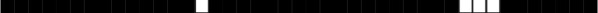
Orphan	9	EA	T	557347777740261	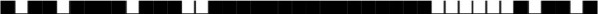
Orphan	9	EA	T	557307777740261	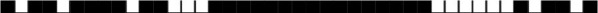
Orphan	9	EA	T3-ETH	557000377740261	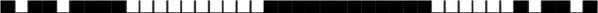
Orphan	9	EAI	CAS1-Kili	513347400003661	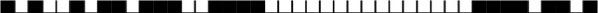
Orphan	8	EAI	CAS1-Delhi	513347740003461	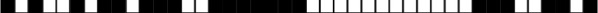
Orphan	6	EA	T3-ETH	555000177740261	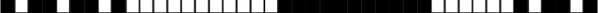
Orphan	5	EA	T	557307077600061	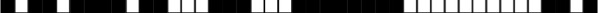
Orphan	4	IO	MANU2	557347437743661	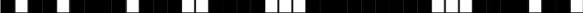
Orphan	4	EA	T3-ETH	577000377740261	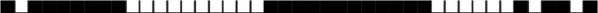
Orphan	4	EA	T3-ETH	577000177740771	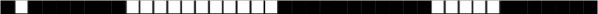
Orphan	3	EA	T	557347437740261	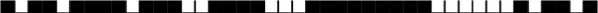
Orphan	3	EA	T	557747437740261	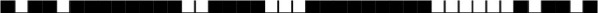
Orphan	2	EA	LAM3	557147407740261	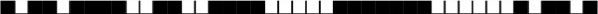
Orphan	2	EA	T	557347777760661	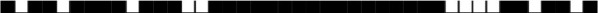
Orphan	2	EAI	Cas1-Delhi	513767740013571	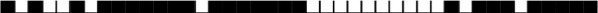
41	2	EA	TURKEY	777777404760771	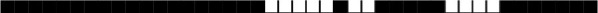
Orphan	2	EA	T3-ETH	517000377740661	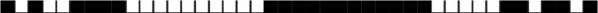
Orphan	2	EA	T3-ETH	517200030740261	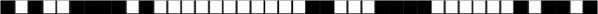
Orphan	2	EA	T	511307437740261	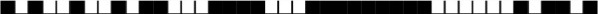
Orphan	2	EA	T	556307737740661	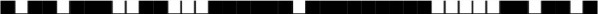
Orphan	2	EA	T	577747777740661	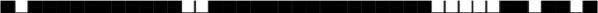
37	2	EA	T3	777737777760771	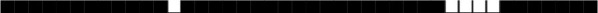
Orphan	2	EA	H3	557347775720661	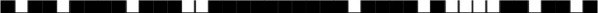
Orphan	2	EA	MANU2	777747777743661	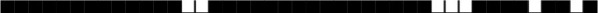
Orphan	2	EA	MANU2	777247777762661	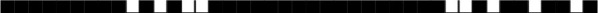
Orphan	2	EA	MANU2	757357777743771	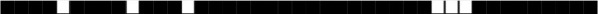
Orphan	2	IO	MANU3	777767777430771	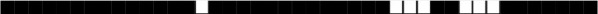

*CBN, conformal Bayesian network; EA, Euro-American; EAI, East-African-Indian; IO, Indo-Oceanic; SIT, spoliogotype international type*.

On the other hand, 150 singleton spoligotypes were detected, which constituted of 47.6% of the total isolates. Majority of the isolates (91.1%) were grouped under orphan spoligotypes whereas only 8.9% were grouped under shared spoligotypes. The isolates were grouped under five major lineages based on the SITVIT2 nomenclature. The identified major lineages included Euro-American (EA), East-African-Indian (EAI), Indo-Oceanic (IO) lineages, *Mycobacterium africanum*, and *Mycobacterium bovis*, accounting for 250 (79.4%), 31 (9.8%), 31 (9.8%), 2 (0.63%), and 1 (0.32%) of the total isolates, respectively.

### Distribution of Dominant Major Lineages and Sublineages Across the Zones of the Study Area

Distribution of the dominant major lineages or sublineages varies across the zones of the study area.

**Figure 2:**
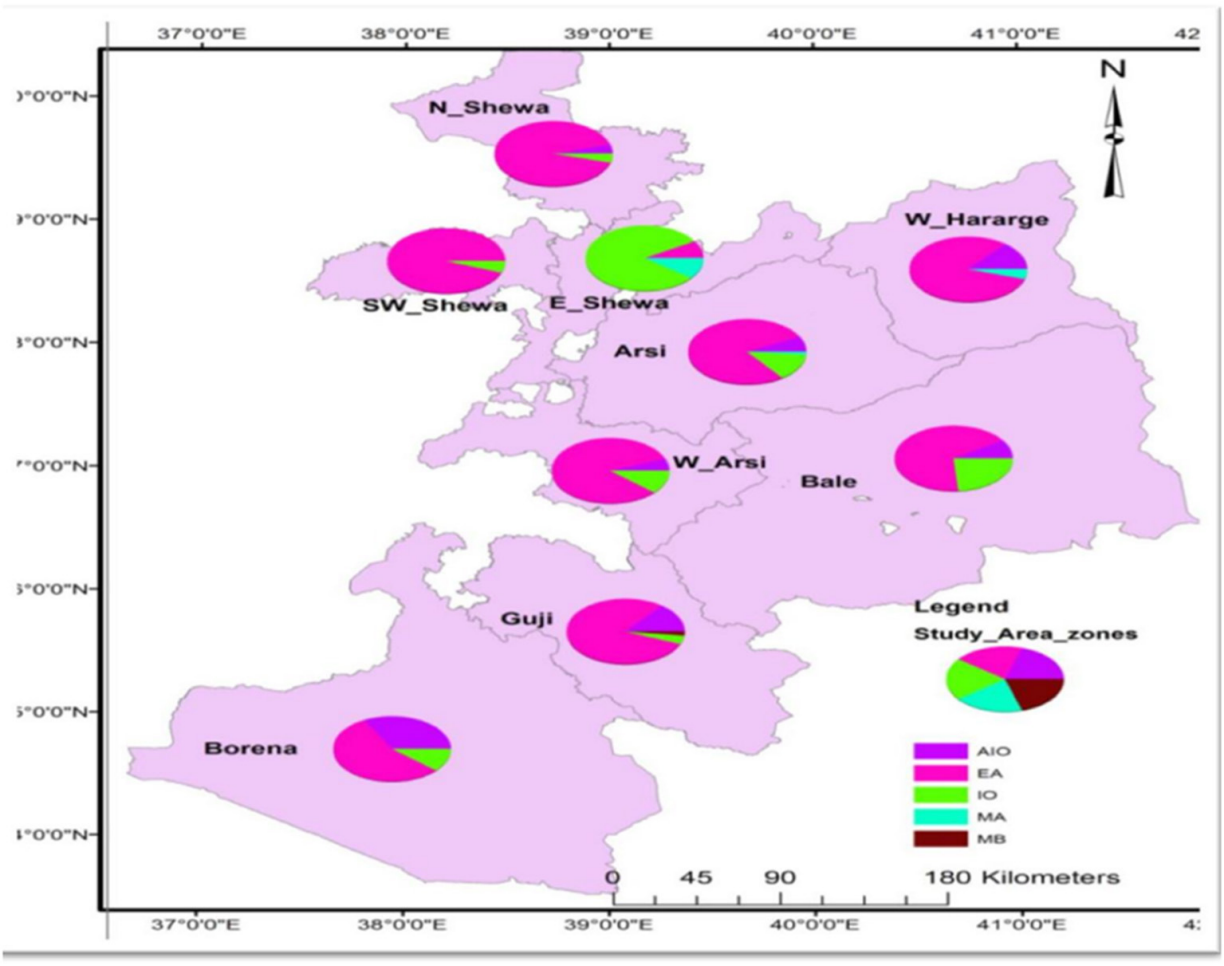
Map showing proportion of major lineages of MTB strains per zones circulating in Oromia, 2020. EAI, East-African-Indian; EA, Euro-American; IO, Indo-Oceanic; MA, *M. africanum*; MB, *M. bovis*; N_Shewa, North Shewa; W_Hararge, West hararge; SW_Shewa, South West Shewa; E_Shewa, East Shewa; W_Aersi, West Aresi.

EA was the dominant major lineage followed by EAI and IO lineages in terms of frequency of occurrence. One case of *M. bovis* was isolated from the Guji Zone from the pastoral community. *M. africanum* was isolated from the Arsi and the West Harerge zones. Members of the EA lineage were widespread in the different zones. As depicted in Figure 2, 55 (22%) and 40 (16%) isolates of the EA lineage were isolated from the Arsi and Guji zones, respectively. T3-ETH and T, the sublineages, were isolated from all of the zones. On the other hand, MANU2 was more prevalent in North Shewa zone than other zones.

### Association of Mycobacterial Lineage With Clustering or Patients' Characteristics

Variation was observed in the clustering rate in the major lineages and dominant sublineages. Nineteen (61.3%) isolates in the EAI lineage grouped in the clustered spoligotypes, which was significantly (*p* < 0.001) higher than the clustering rate of any of the other major lineages. Furthermore, the clustering was significantly (*p* < 0.001) more common in T3-ETH and Manu 3 sublineages than in any of the other sublineages (Table 3). The binary logistic regression model revealed association of EA lineage with age (19, 29–38) (*p* < 0.05), Guji zone (*p* < 0.05), retreatment cases (*p* < 0.05), and the absence of history of BCG vaccination (*p* < 0.05). However, the association of EA lineage observed in binary logistic regression was not observed (*p* > 0.05) when multiple logistic regression model analysis was applied (Table 4).

**Table 3:** Comparison of clustering status within groups of selected variables (*n* = 315).

**Characteristics**		**Total**	**Clustered**	**Nonclustered**	***P*-value**
		***N* (%)**	***N* (%)**	***N* (%)**	
Zones	North Shewa	24 (7.6)	12 (50.0)	12 (50.0)	0.126
	South-West Sh	18 (5.7)	9 (50.0)	9 (50.0)	
	East Shewa	61 (19.4)	32 (52.5)	29 (47.5)	
	Arsi	70 (22.2)	31 (44.3)	39 (55.7)	
	West Arsi	18 (5.7)	11 (61.1)	7 (38.9)	
	Bale	34 (10.8)	14 (41.2)	20 (58.8)	
	Guji	50 (15.9)	34 (68.0)	16 (32.0)	
	Borena	18 (5.7)	7 (38.9)	11 (61.1)	
	West Harerge	22 (7.0)	15 (68.2)	7 (18.2)	
Major lineages	Euro-American	250 (79.4)	140 (56.0)	110 (44.0)	0.001^*^
	East-African-Indian	31 (9.8)	19 (61.3)	12 (38.7)	
	Indo-Oceanic	31 (9.8)	6 (19.4)	25 (80.6)	
	*M. africanum*	2 (0.63)	0 (0)	2 (100)	
	*M. bovis*	1 (0.32)	0 (0)	1 (100)	
Sublineage	T3-ETH	76 (24.1)	65 (85.5)	11(14.5)	0.001
	T	70 (22.2)	35 (50.0)	35 (50.0)	
	Manu2	36 (11.4)	22 (61.1)	14 (38.9)	
	CAS1-Delhi	17(5.4)	10 (58.8)	7 (41.2)	
	CAS1-Killi	17 (5.4)	9 (52.9)	8 (47.1)	
	H3	17 (5.4)	2 (11.8)	14 (88.2)	
	Manu3	13 (4.1)	11 (84.6)	2 (15.4)	
	X2	10 (3.2)	1 (10)	9 (90.0)	
	Others	59 (18.7)	10 (16.9)	49 (83.1)	

* *Fisher's exact test*.

**Table 4:** Association of patients' socio-demographic characteristics and clinical history with Euro-American lineage compared to other major lineages (*n* = 315).

**Patient**	**Euro-American**	**COR, 95% CI**	***P-value***	**AOR, 95%CI**	***P-value***
**characteristics**	**lineage [*N*(%)]**				
Age (29–39)	57 (53.77)	2.035, 1.053–3.934	0.035	2.229, 1.057–4.999	0.36
Zone	40 (66.67)	2.933, 0.985–8.733	0.053	0.868, 0.319–2.366	0.783
Retreatment cases	62 (57.94)	0.695, 0.558–1.161	0.144	1.655, 0.791–3.403	0.181
BCG not vaccinated	174 (54.04)	2.736, 1.239–6.040	0.013	0.391, 0.150–1.022	0.055
BMI (abnormal)	136 (57.38)	0.718, 0.416–1.242	0.237	0.546, 0.270–1.105	0.092

*COR, crude odds ratio; AOR, adjusted odds ratio; CI, confidence interval; BCG, Bacille Calmette–Guerin; BMI, body mass index*.

## Discussion

In this study, MTBC was isolated from patients with TB in nine zones of the Oromia region, and the isolates were spoligotyped. Spoligotyping is most commonly used for genotyping for mycobacteria in the country (34, 35). The study identified SIT, sublineages, and major lineages of the members of MTBC. EA, EAI, and IO were the three most frequently isolated lineages of *M. tuberculosis*, which is in agreement with the results of the previous studies conducted in the country (19, 20, 36–38). On the other hand, *M. bovis* was the least isolated species in the Oromia region, as only one isolate was confirmed, out of the 315 isolates. Similar observations were made by earlier studies done in the country (17, 21, 22). Thus, the findings of both this study and previous studies could suggest that the role of *M. bovis* in causing human TB in Ethiopia is low. The reason may be associated with the low prevalence of bovine TB in the zebu cattle that is kept under extensive traditional farming.

The predominant sublineage isolated in the study area was T3-ETH that was unique to Ethiopia (18) whereas the dominant spoligotype identified was SIT149, which belongs to lineage 4. This observation agrees with the findings of several studies reported from different parts of the country (17, 39, 40). The T sublineage was the second dominant sublineage that was reported by this study and other studies from Ethiopia (19, 36, 37). An earlier study indicated that the occurrence of the T sublineage is influenced with geographic location (31). Furthermore, these sublineages were also reported from other African countries and other continents (41). The reason could be due to the movement of *M. tuberculosis*-infected people from place to place crossing international boundaries for socioeconomic reasons, displacement for political unrest, or natural disasters.

The finding of significant number of orphan types could be due to the lack of previous reports from the Oromia region whereas the large number of singletons in this study might be due to the wide geographical coverage of the study area that can lead to the detection of many different new spoligotypes. In addition, the low discriminatory power of spoligotyping could result in detection of several orphan types and singletons as the isolates could not be differentiated to a maximum degree. In addition, spoligotyping is sensitive and error-prone particularly during the interpretation of its results. The interpretation of the result of spoligotyping is based on the subjective judgment in classifying the binary values (the absence and the presence of spacers). Therefore, spoligotyping should be supported by techniques with a better discriminating power, such as DNA sequencing, that allows identifying true phylogenetic relationships, so that the results can be more acceptable (42).

The result of this study showed an overall clustering rate of 52.4%, which is lower than that reported by the Ethiopian national survey, the Afar region and central Ethiopia, which were 70% (40), 76.2% (22), and 79.3% (20), respectively. But, it was higher than the clustering rate reported from northwest Ethiopia was 45.1% (18). The variation in clustering rate in different regions of Ethiopia could be due to differences in the population density of the study areas, socioeconomic status of the study subjects, the mobility of the population of the study areas (39), and effectiveness of TB control programs. In this study, clustering was more common in EAI lineage and T3-ETH sublineage, suggesting their potential in spreading effectively and causing infection in the country (43).

The RTI was 0.42 in this study, and it was less than that reported from central Ethiopia and the Afar region, which were 0.8 (20) and 0.58 (22), respectively. But, it was greater than that reported from the Amhara region that was 0.3 (18). The variation in RTI values of different studies is associated with the factors that affect clustering, including the efficiency of the TB control program, virulence of the strain of *M. tuberculosis*, socioeconomic status of the population, and other related factors of these study areas. The values of RTI lie between 0 and 1 where 0 indicates the least threat of epidemics whereas values greater than 0 approaching 1 (100%) indicate different levels of epidemics, which necessitates interventions. However, the use of RTIs to calculate the recent transmission rate has a major limitation because RTIs do not consider the diversity of strains and mutation (44). Thus, the interpretation of RTI is affected by the genetic factors of the pathogen, epidemiological links between hosts, and public health interventions (45). Nonetheless, in the case of this study, since spoligotyping was used for genotyping, the RTI for the assessment of recent transmission can be suboptimal and considered as a limitation.

The poor discriminatory power of spoligotyping is associated with the inherent genetic marker based on which it was developed that makes it prone to homoplasy. The use of finite numbers of sequences as a genotyping marker named clustered regulatory short palindromic repeats (CRISPRs) in spoligotyping leads to the occurrence homoplasy (46). Comas et al. (47) evaluated the performance of spoligotyping on 97 MTBC strains by considering multilocus sequence analysis (MLSA) as a gold standard. Their finding indicated that soligotyping could not detect five of the seven main strain lineages as monophyletic groupings. In addition, the analysis of the 97 MTBC strains revealed that the phylogenies derived from spoligotyping was significantly incompatible with the MLSA data. This significant incongruence between spoligotyping and the MLSA data was because of homoplasy. Thus, based on these data, the authors concluded that using spoligotyping to define deep phylogenetic groupings in MTBC cannot produce reliable results (47–49). By contrast, DNA sequencing allows to identify true phylogenetic relationships, and to discover single-nucleotide polymorphisms (SNPs) that can be used as powerful genotyping markers (50). However, DNA sequencing is likely to remain limited to specialized sequencing centers for some time. Therefore, generating genotyping data for local epidemiology and broader applications in monomorphic microbes, such as MTBC, will remain challenging. Comas and his coauthors (47) suggested to combine spoligotyping and mycobacterial interspersed repetitive units (MIRUs)-based variable number tandem repeats (VNTRs) typing use for initial exploratory screening of strains.

## Conclusion

The predominant isolation of SIT149 and EA lineage from the Oromia region substantiates the findings of similar studies that are conducted in Ethiopia in other regions of Ethiopia. The observation of a significant number of orphans spoligotypes could suggest the lack of prior similar reports in the study area warranting for further studies using DNA sequencing that allows identifying true phylogenetic relationships.

## Data Availability Statement

The raw data supporting the conclusions of this article will be made available by the authors, without undue reservation.

## Ethics Statement

The studies involving human participants were reviewed and approved by Health Studies Higher Degree Committee of University of South Africa (Ref Nos. REC-012714-039 and HSHDC/454/2015) and the Oromia Health Bureau, Ethiopia, permitted the field work in the study area (Ref No. BEFO/AHBTM/1-8/2308). The patients/participants provided their written informed consent to participate in this study.

## Author Contributions

BH was involved in the conception, design, acquisition of the data, statistical analysis, interpretation of the data, and drafting the manuscript. AZ was involved in data acquisition and critical revision of the manuscript. BW and AH were involved in the analysis, interpretation of the data, and critical revision of the manuscript. GA was involved in the design, guiding the data collection, interpretation of the result, and critical revision of the manuscript. All authors approved the manuscript for publication and agreed to be accountable for all aspects of the work done.

## Funding

This study was conducted by the small financial support that the first author received from the Mede-Walabu and Addis Ababa Universities.

## Conflict of Interest

The authors declare that the research was conducted in the absence of any commercial or financial relationships that could be construed as a potential conflict of interest.

## Publisher's Note

All claims expressed in this article are solely those of the authors and do not necessarily represent those of their affiliated organizations, or those of the publisher, the editors and the reviewers. Any product that may be evaluated in this article, or claim that may be made by its manufacturer, is not guaranteed or endorsed by the publisher.

## Abbreviations

ALIPB: Aklilu Lemma Institute of Pathobiology
DNA: deoxyribonucleic acid
CRISPR: clustered regulatory short palindromic repeats
EA: Euro-American
AEI: East-African-Indian
FQ: fluoroquinolones
IO: Indo-Oceanic
LJ: Lowenstein Jenson
MLSA: multilocus sequence analysis
MTBC: *Mycobacterium tuberculosis* complex
MIRUs: mycobacterial interspersed repetitive units
NTM: nontuberculosis mycobacteria;
PCR: polymerase chain reaction
pre-XDR TB: preextensively drug-resistant TB
RTI: recent transmission index
SNP: single-nucleotide polymorphisms
SIT: Spoligotype international type
TB: tuberculosis
XDR-TB: extensive drug-resistant TB
VNTRs: variable number tandem repeats
ZN: Ziehl–Neelsen.
